# NGLY1 Deficiency, a Congenital Disorder of Deglycosylation: From Disease Gene Function to Pathophysiology

**DOI:** 10.3390/cells11071155

**Published:** 2022-03-29

**Authors:** Ashutosh Pandey, Joshua M. Adams, Seung Yeop Han, Hamed Jafar-Nejad

**Affiliations:** 1 Department of Molecular and Human Genetics, Baylor College of Medicine, Houston, TX 77030, USA; josada@med.umich.edu (J.M.A.); seungyeop.han@bcm.edu (S.Y.H.); 2 Program in Developmental Biology, Baylor College of Medicine, Houston, TX 77030, USA; 3 Department of Pediatrics, University of Michigan, Ann Arbor, MI 48109, USA; 4 Genetics & Genomics Graduate Program, Baylor College of Medicine, Houston, TX 77030, USA; 5 Development, Disease Models & Therapeutics Graduate Program, Baylor College of Medicine, Houston, TX 77030, USA

**Keywords:** *N*-glycosylation, deglycosylation, congenital disorder of deglycosylation (CDDG), proteasome, mitochondrial abnormality, BMP signaling, AMPK signaling, ER-associated degradation (ERAD), rare disease, human developmental disorder

## Abstract

*N*-Glycanase 1 (NGLY1) is a cytosolic enzyme involved in removing *N*-linked glycans of misfolded *N*-glycoproteins and is considered to be a component of endoplasmic reticulum-associated degradation (ERAD). The 2012 identification of recessive *NGLY1* mutations in a rare multisystem disorder has led to intense research efforts on the roles of NGLY1 in animal development and physiology, as well as the pathophysiology of NGLY1 deficiency. Here, we present a review of the NGLY1-deficient patient phenotypes, along with insights into the function of this gene from studies in rodent and invertebrate animal models, as well as cell culture and biochemical experiments. We will discuss critical processes affected by the loss of NGLY1, including proteasome bounce-back response, mitochondrial function and homeostasis, and bone morphogenetic protein (BMP) signaling. We will also cover the biologically relevant targets of NGLY1 and the genetic modifiers of NGLY1 deficiency phenotypes in animal models. Together, these discoveries and disease models have provided a number of avenues for preclinical testing of potential therapeutic approaches for this disease.

## 1. Introduction

**Figure 1:**
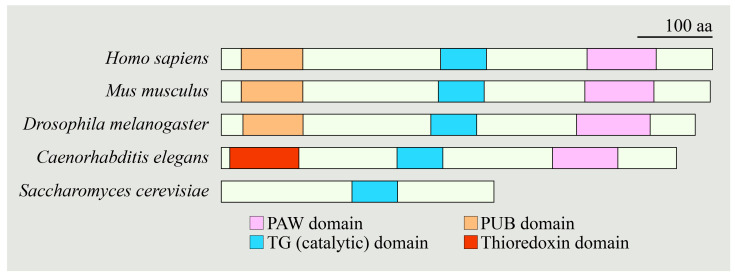
Schematic representation of *N*-glycanase 1 orthologs with domains present in the indicated species. aa, amino acid; PAW, a domain in PNGases and other worm proteins; PUB, a domain in PNGase/UBA or UBX-containing proteins; TG, transglutaminase-like.

Thousands of proteins in the secretory pathway of eukaryotic cells are *N*-glycosylated, which is a co-translational and post-translational modification critical for proper folding and function of many proteins [1]. During the protein quality control process, some proteins fail to fold properly in the endoplasmic reticulum (ER) and are therefore routed towards proteasomal degradation via the ER-associated degradation (ERAD) pathway [2,3]. At least some proteins undergo de-*N*-glycosylation, i.e., removal of *N*-linked glycans from the misfolded protein, as part of this process. *N*-Glycanase 1 (NGLY1), which is called peptide: *N*-glycanase (PNGase) or a variation of “PNG/Png” in some organisms, is a de-*N*-glycosylating enzyme first identified in yeast [4]. Later, its homologs were characterized in other organisms, including mammals and non-mammals. The human NGLY1 protein has three major domains: an *N*-terminal ubiquitin-binding domain (Peptide: *N*-glycanase/UBA or UBX-containing proteins; PUB), a catalytic core domain, and a C-terminal carbohydrate-binding domain (PAW) [5]. The PAW domain binds to high-mannose structures on the retrotranslocated or misfolded glycoproteins, whereas the PUB domain interacts with various ERAD components, such as valosin-containing protein (VCP/p97), Rad23, and Derlin-1 (DERL1) [6,7,8,9,10]. Figure 1 shows a schematic representation of the NGLY1 protein domains across several species.

Recessive mutations in human *NGLY1* lead to a disorder called NGLY1 deficiency (OMIM# 615273), which presents with multiple phenotypes, including but not limited to global developmental delay, movement disorder, seizures, microcephaly, liver disorders, and chronic constipation [11,12,13,14]. It is categorized as the first known congenital disorder of deglycosylation (CDDG) [15]. Work in a number of laboratories has provided insight into the biological functions of NGLY1 and has identified several downstream proteins and signaling pathways affected by the loss of NGLY1 in model organisms and mammalian cells. These studies have paved the way for designing and testing potential therapeutic strategies in preclinical models and for conducting small-molecule and drug-repurposing screens for NGLY1 deficiency. Moreover, they have shed light on novel regulatory mechanisms for processes such as proteasomal degradation, developmental signaling, and mitochondrial homeostasis, which are affected in common diseases as well.

Although there are no approved treatments for NGLY1 deficiency at this time, NGLY1 research has gained significant momentum in recent years. In this review, we covered the findings from clinical, as well as model organism- and cell-based studies, towards understanding the roles of NGLY1 in animal development and physiology. We hope that this work will stimulate further research on this fascinating gene and will thereby contribute to the development of therapeutic options for NGLY1 deficiency.

## 2. Patient Phenotypes in NGLY1 Deficiency

### 2.1. NGLY1 Deficiency Phenotypic Presentations Are Diverse

**Figure 2:**
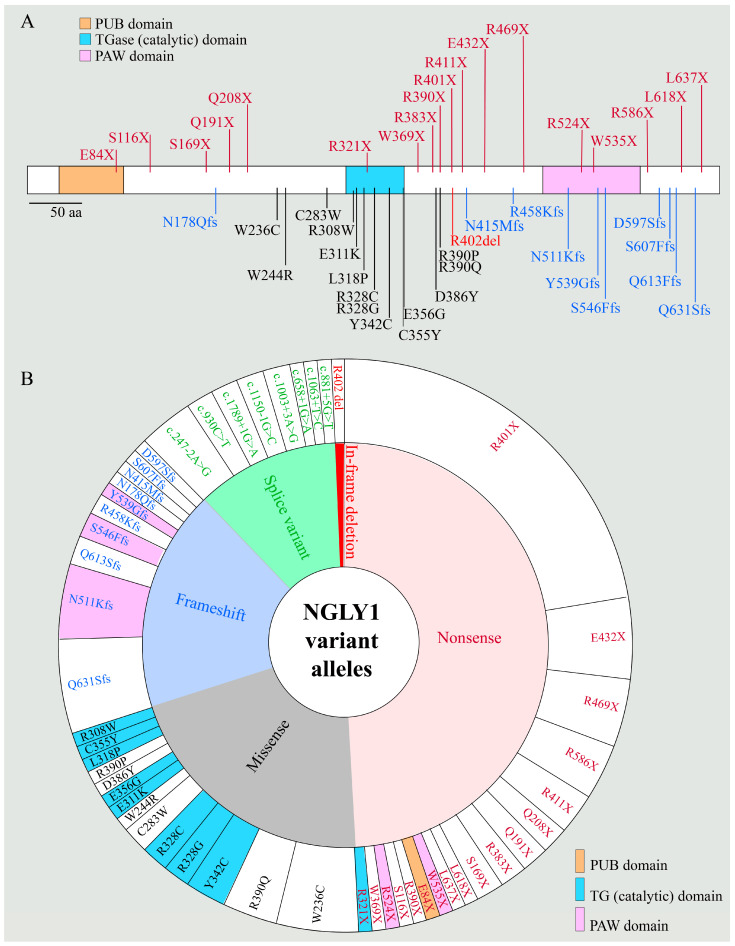
(**A**) Schematic representation of the reported missense, nonsense, frameshift, and in-frame deletion variants in *NGLY1*. Nonsense variants are in maroon font above the NGLY1 schematic. Missense (black font), frameshift (blue font), and in-frame deletion (red font) are shown below NGLY1. Protein structure is based on SMART (simple molecular architecture research tool; http://smart.embl-heidelberg.de accessed on 21 March 2022). (**B**) Ring chart diagram showing the reported variants, including splice site variants (green font) with their relative occurrence. Note that R401X is the most common variant (~21%). aa, amino acid; fs, frameshift; PAW, a domain in PNGases and other worm proteins; PUB, a domain in PNGase/UBA or UBX-containing proteins; TG, transglutaminase-like.

Damaging variants in both copies of *NGLY1* were first reported by the Goldstein group in 2012 in a patient with an undiagnosed, multisystem developmental disorder [14]. The patient was noted to have features like those found in congenital disorders of glycosylation (CDG), including developmental delay, epilepsy, choreoathetosis, liver dysfunction, and alacrima (lack of tears). On liver biopsy, the authors observed an unidentified material throughout the cytoplasm, compatible with the suggested role for NGLY1 in the degradation of misfolded glycoproteins. Identification of a second patient with similar phenotypes and harboring *NGLY1* variants in 2013 established NGLY1 deficiency as a new rare genetic disorder [16]. Since then, more than 100 patients with this disorder have been identified [17], thanks in no small part to the efforts of the first two families in raising awareness about this disease, as well as advances in and the growing accessibility of next-generation sequencing technologies around the world. More than 60 of these patients have already been formally reported in one or more publications [13]. Figure 2 shows the types of variants reported in patients with NGLY1 deficiency and the location of those that affect the protein sequence on a schematic of the NGLY1 proteins. Table 1 lists the reported patients, along with their gender, age at the time of publication, genotype at the *NGLY1* locus, reported phenotypes, and reference(s). Although the initial clinical triad of hypo/alacrima, choreoathetosis, and elevated liver transaminases has been observed in subsequent patients [18,19], additional findings have complicated the picture, indicating that NGLY1 deficiency affects more organ systems than originally described [11,12,20,21,22].

The first cohort of patients with NGLY1 deficiency was described by Enns and colleagues in 2014 and included the first two patients, as well six additional patients who showed symptoms and signs similar to those observed in the first two patients, including athetosis, hypotonia, epilepsy, liver dysfunction, and alacrima [11]. Despite phenotypic similarities between NGLY1 deficiency and CDGs, analysis of this cohort clarified that the overall phenotypic presentation of NGLY1 deficiency is distinct from that of CDGs; for example, NGLY1 deficiency does not seem to cause cerebellar atrophy, lipodystrophy, or significant cardiac phenotypes, all of which are present in many CDGs [11,36].

In addition to confirming the key features of NGLY1 deficiency, this report expanded the clinical phenotype. Some patients were noted to have peripheral neuropathy. Moreover, the observed increase in transaminases proved to be transient in many patients. In one patient, the symptoms developed slowly over infancy, suggesting that NGLY1 deficiency may have a variable disease presentation in addition to being a progressive condition [11]. This has been noted in several patients in subsequent publications [24,37]. The authors also noted that three patients were found to have adrenal cortex vacuolation, including two deceased patients [11]. This was the first clue for a potential adrenal insufficiency in patients with NGLY1 deficiency. The authors also connected the clinical features with the role of NGLY1 in the ERAD pathway, noting similarities to conditions caused by mutations in this pathway.

Despite the presence of adrenal cortex vacuolation in a subset of patients with NGLY1 deficiency, their adrenal function had not been evaluated at the time of Enns’ report [11]. However, in 2019, van Keulen et al. described the case of an 8-year-old girl who was noted to have recurrent respiratory infections, vomiting, and bronze skin tinting, in addition to the classic findings of NGLY1 deficiency [27]. The patient had low cortisol with high adrenocorticotropic hormone levels, and no adrenal glands were found on ultrasound. These observations, together with additional laboratory exams, confirmed a diagnosis of adrenal insufficiency. Indeed, her symptoms were improved upon hormone replacement therapy [27]. The authors connected their findings with the previous findings of adrenal cortex vacuolation and low unconjugated estriol and raised the possibility that the unexplained death reported in some children with NGLY1 deficiency might have resulted from adrenal insufficiency. Therefore, it stands to reason to evaluate adrenal function in patients with NGLY1 deficiency as van Keulen et al. recommend [27], especially given that adrenal insufficiency is treatable and therefore a potentially preventable cause of demise in these patients.

In Enns’ original manuscript, all three patients who were examined for the function of peripheral neurons were noted to have peripheral neuropathy [11]. A year later, Caglayan and colleagues described two patients with developmental delay at 6 months with loss of skills and corneal opacity, both of whom also had peripheral neuropathy [24]. Nerve conduction findings suggested a polyneuropathy, and sural nerve biopsy in an 8-year-old patient confirmed diffuse axonal loss [24]. In 2017, Lam et al. reported on 11 patients, with nerve conduction studies demonstrating an axonal sensorimotor polyneuropathy with demyelination [12]. Together, these reports establish neuropathy as a major phenotype in NGLY1-deficient patients. The molecular basis for the neurological phenotypes observed upon loss of *NGLY1* is not known. Of note, previous work in *C. elegans* demonstrated a role for NGLY1 in axonal branching, suggesting a potential mechanism for the neuropathy observed in human patients [38]. Together, the patient and worm data highlight the importance of NGLY1 in axonal development and/or maintenance.

Liver dysfunction was reported in the first-described patient with NGLY1 deficiency [14]. Over the past several years, this phenotype has been better elucidated. Heeley et al. noted the presence of liver cirrhosis in their patient aged 6 months, with accumulated intracytoplasmic material similar to Need’s initial report [14,25]. Rios-Flores and colleagues later described a patient with reversible acute liver failure distinct from the transient liver enzyme elevations previously described [33]. Lipiński et al. described a child with NGLY1 deficiency who had significantly elevated transaminases at 10 months of age. A liver biopsy of this patient at three years of age showed steatosis and fibrosis with amorphous material in the cytoplasm [31]. Moreover, Lipari Pinto and colleagues reported another patient with persistent elevation of liver transaminases [34]. These clinical findings emphasize the potential importance of NGLY1 in liver development and/or hepatocyte function and make the liver function an important clinical measure to monitor in patients.

Several additional phenotypes have been noted over the past several years but are not yet widely reported. Bosch and colleagues were the first to ascribe NGLY1 deficiency as a cause of cerebral visual impairment (CVI) [26]. CVI had previously been associated with CDG, and in a study of patients with CVI of unknown genetic origin, the authors identified one patient with NGLY1 deficiency. Further work is necessary to better understand the prevalence of CVI in patients with NGLY1 deficiency and its pathophysiological mechanisms. Cerebral atrophy, noted in multiple reports, may be a cause, but this is not yet well understood. In 2021, Dabaj and colleagues reported a patient with the classic findings of NGLY1 deficiency, along with CVI and a ventricular septal defect (VSD) [35]. This was the first study to identify a cardiac phenotype in a patient with NGLY1 deficiency [35], although VSDs have also been observed in *Ngly1*-deficient mouse embryos ([39,40], see below).

### 2.2. Biomarkers of NGLY1 Deficiency

In recent years, there has been an effort to identify biomarkers that may help predict the severity of NGLY1 deficiency, as well as alert providers of the need for genetic workup. Lam et al. utilized magnetic resonance imaging (MRI) and in vivo magnetic resonance spectroscopy (MRS) analysis of the brain to identify potential changes on imaging as a function of patient age or disease worsening [12]. Cerebral atrophy was noted in six of nine patients, showed a correlation with worse function, and was progressive in one patient with follow-up MRI [12]. MRS showed changes in several metabolites (including some amino acids) in specific brain regions, which again showed a correlation with an increase in age and a decline in brain volume and function [12]. Cerebrospinal fluid (CSF) analysis showed the levels of neurotransmitter metabolites 5-HIAA, HVA, and BH4 to be low in most patients. Importantly, the levels of these metabolites were strongly correlated with cerebral atrophy on imaging.

Hall and colleagues utilized matrix-assisted laser desorption/ionization time of flight mass spectrometry (MALDI-TOF MS) to identify a urinary-excreted metabolite (Neu5Ac-Hex-GlcNAc-Asn) in NGLY1-deficient patients [41]. Chang et al. later identified elevations in plasma methionine, plasma S-adenosylmethionine (SAM), and plasma S-adenosylhomocysteine (SAH) in a 5-year-old female with NGLY1 deficiency, which remained elevated for at least one year following the initial assessment [29]. The same year, Haijes et al. identified aspartylglycosamine (GlcNAc-Asn) from dried blood spots as a potential biomarker for this disease [30]. Of note, GlcNAc-Asn is also significantly increased in both plasma and urine of a mouse model of NGLY1 deficiency [42]. These biomarkers offer the potential to better screen patients, as well as in tracking treatment efficacy in future clinical trials. In fact, Mueller and colleagues have recently established a quantitative assay for measuring GlcNAc-Asn levels in biological samples and have shown that GlcNAc-Asn is a reliable marker for loss of NGLY1 in cell lines, rat tissues, and urine and plasma from both *Ngly1*^−/−^ rats and NGY1-deficient patients [17].

## 3. Animal Models of NGLY1 Deficiency

### 3.1. Mammalian Models

Developing a reliable mammalian model for NGLY1 deficiency is critical to identifying and testing potential treatment modalities. Several studies over the past few years have attempted to characterize mammalian models of NGLY1 deficiency in relationship to the disease phenotypes. The first study of an *Ngly1*-deficient mouse model was published by the Suzuki group in 2017, following their earlier work on *Ngly1*-mutant mouse embryonic fibroblasts (MEFs) established from these mice [39,43]. This allele, which is officially called *Ngly1^tm1^.^1Tasuz^*, lacks exons 11 and 12 and shows homozygous lethality at late embryonic stages on a C57BL/6 genetic background [39]. Of note, all mutant embryos were found to have ventricular septal defects at E16.5, along with partially penetrant anemia and edema. Because cardiac findings had not been observed in NGLY1-deficient patients at the time of publication, it was not clear how closely the loss of *Ngly1* in mice will recapitulate the human phenotypes. However, as mentioned above, Dabaj et al. have recently reported a patient with the classic features of NGLY1 deficiency, along with a ventricular septal defect, suggesting that cardiac findings may indeed be a rare feature of patients with NGLY1 deficiency [35].

Whole-exome sequencing has led to the identification of many loss-of-function variants in *NGLY1*, including missense, frame shift, nonsense, and splice site variants (Figure 2A,B). Analysis of the phenotypes reported for patients with these variants does not as of yet show a clear genotype–phenotype correlation in NGLY1-deficient patients (Table 1), suggesting that genetic background might play a key role in *NGLY1* loss-of-function phenotypes. Indeed, the authors of the above-mentioned study reported that on a C57xICR mixed background, around half of animals homozygous for their *Ngly1* allele survived beyond the embryonic period [39]. The surviving animals showed rather severe phenotypes. Phenotypic onset occurred in the first few weeks of life, with only 30% survival at 3 weeks of age. More recently, the Suzuki lab assessed the effects of the Japanese-fancy mouse (JF1) genetic background on *Ngly1* loss-of-function phenotypes [42]. The JF1 *Ngly1*^−/−^ animals survived to at least 18 months, although they developed a progressive hindlimb clasping, rotarod decline, and reduced grip strength starting at 4 weeks of age. The authors noted the accumulation of ubiquitinated proteins in the thalamus and spinal cord. Although there was no evidence of central nervous system (CNS) neurodegenerative lesions, progressive axonal atrophy was noted similar to that seen in patients with NGLY1 deficiency. Finally, the mutant mice showed an increase in GlcNac-Asn in the urine like that seen in NGLY1-deficient patients, suggesting that this biomarker is conserved between humans and mice and could be used as a marker of phenotypic progression in preclinical therapeutic trials [30]. Altogether, these studies provide strong evidence that genetic background can exert a profound effect on the presentation and progression of NGLY1 deficiency phenotypes.

In recent years, several groups have used a second loss-of-function allele of *Ngly1* called *Ngly1^em4Lutzy^* in mechanistic studies. This allele is thought to be a functional null allele and also shows late embryonic/perinatal lethality [44]. It was generated by the Lutz group at Jackson laboratory using CRISPR/Cas9 genome editing and harbors an 11-bp deletion in exon 8, which results in a frame-shift mutation. Similar to the *Ngly1^tm1.1Tasuz^* allele, *Ngly1^em4Lutzy^* is homozygous lethal on a C57BL/6 background. The high degree of embryonic lethality in *Ngly1*^−/−^ mice on a C57BL/6 background initially complicated the study of patient phenotypes and cast doubt on the utility of mouse as a model for preclinical therapeutic studies for NGLY1 deficiency. Fortunately, the partial suppression of *Ngly1*^−/−^ lethality on mixed genetic backgrounds has offered promising models for both mechanistic and preclinical studies on this disease.

One of the more consistent findings in patients is liver dysfunction, which is often transient and sometimes associated with accumulation of unidentified cytoplasmic material [11,14]. Using a liver-specific loss of *Ngly1* (*Albumin-Cre; Ngly1^floxflox^*) as a model, Fujihira et al. observed normal animal survival with no obvious phenotype, indicating that liver dysfunction does not contribute to embryonic lethality of *Ngly1*^−/−^ animals [45]. Electron microscopy revealed age-dependent abnormalities in hepatocyte nuclear size and morphology, and showed dilated bile canaliculi, which is suggestive of a biliary excretion defect. When fed with a regular diet, *Albumin-Cre; Ngly1^floxflox^* animals did not show any functional liver defects. However, feeding with high-fructose, high-fat, or methionine-choline-deficient diets resulted in steatohepatitis in these animals. The high-fructose diet, which is used to generate a non-alcoholic fatty liver disease model, also led to increased liver transaminases and decreased albumin levels, suggestive of impaired liver function. As illustrated by this example, generation of the *Ngly1^flox/flox^* strain by the Suzuki group offers a valuable resource for studying the role of NGLY1 in specific tissues affected in NGLY1-deficient patients.

A major advancement in animal models came in 2020, when Asahina and colleagues developed a rat model of NGLY1 deficiency that demonstrated phenotypes similar to those in human patients [46]. Using the CRISPR/Cas9 technology, the investigators introduced a ~2.6-kb deletion in the *Ngly1* locus, which removes its last two exons (11 and 12) and extends into the 3′ flanking region of *Ngly1*. The heterozygous rats were viable and fertile and did not show any gross morphological phenotypes. However, starting from a young age, rats deficient for *Ngly1* (*Ngly1^−/−^*) showed progressive hindlimb clasping, reduced grip strength, and rotarod test defects, suggestive of impaired motor function. The authors also noted impaired spatial learning in *Ngly1^−/−^* rats. Importantly, the authors were able to connect the phenotypes with histopathological findings compatible with neurodegeneration in some parts of the CNS. Defects in the ventral posteromedial, ventral posterolateral, and ventral lateral nuclei of the thalamus were noted. Necrotic lesions and eosinophilic inclusion bodies were also found in the spinal cord and the pons. Notably, although NGLY1 is ubiquitously expressed in the brain, only some brain areas were affected in *Ngly1^−/−^* rat brains, suggesting selective vulnerability of specific brain areas to the loss of deglycosylation. Anti-NeuN staining demonstrated neuronal loss in the thalamus of *Ngly1^−/−^* rats at both time points analyzed (5 and 29 weeks of age). In contrast, examination of the sciatic nerve showed an age-dependent axonal degeneration in these animals. Together with the JF1 *Ngly1^−/−^* and *Ngly1* conditional knockout animals with various Cre lines, the rat model opens the potential for a better understanding of the complex phenotypic presentation observed in patients with NGLY1 deficiency.

Survival of *Ngly1^−/−^* rats beyond the embryonic period and identification of progressive neurological features in them associated with histopathology in the nervous system suggest these animals to be a valuable model for preclinical studies on NGLY1 deficiency. Indeed, Asahina and colleagues have recently reported the utilization of *Ngly1^−/−^* rats to develop a therapeutic measure for the reversal of their neurological phenotypes [47]. The authors generated a recombinant adeno-associated virus serotype 9 (AAV9) vector expressing the human *NGLY1* cDNA under the constitutive CMV promoter (AAV9-hNGLY1). CNS-restricted delivery of AAV9-hNGLY1 in *Ngly1^−/−^* rats improved the motor function and reduced neuroinflammation in these animals. The data provide clear evidence that the neurological phenotypes associated with loss of NGLY1 are at least in part reversible and offer an exciting framework for therapeutic intervention in NGLY1-deficient patients [47]. Therapeutic introduction of a protein in patients with loss-of-function mutations through enzyme replacement therapy, gene therapy, or gene correction runs the risk of eliciting an immune response to the introduced protein, as it can be perceived as “foreign” by the patient’s immune system [48]. In this regard, a recent report on the detection of low levels of the NGLY1 protein in lymphoblastoid cells isolated from multiple NGLY1-deficient patients is welcome news, as it might suggest a lower chance for an immune response upon such therapeutic strategies in these patients [49].

### 3.2. Non-Mammalian Models

In addition to mammalian model organisms, non-mammalian models, such as yeast, *C. elegans*, and *Drosophila* have provided important insights into NGLY1 biology [4,50,51]. The gene encoding a PNGase was first characterized in budding yeast *Saccharomyces cerevisiae* as *PNG1* and found to be evolutionary conserved in eukaryotes [4]. *Saccharomyces cerevisiae* has a single ortholog of NGLY1 (PNGase 1 or Png1). Loss of Png1 in yeast did not exert any overt phenotypes. A PNGase mutant in *Neurospora crassa*, a filamentous fungus, displayed a noticeable hyphal growth defect, the underlying cause of which is not known [52]. Of note, two of the three amino acids directly involved in the catalytic activity of NGLY1 are not present in the *Neurospora* PNGase, strongly suggesting that it regulates hyphal growth via a non-enzymatic mechanism [5,53]. Accordingly, it is possible that NGLY1 plays enzyme-independent roles in other organisms as well.

*Drosophila* has a single homolog of the human NGLY1 called PNGase-like (Pngl), which shares a high degree of structural similarity with human NGLY1 [54]. It has a core transglutaminase-like (TG) domain with a catalytic site, a PUB domain, and a C-terminal carbohydrate-binding PAW domain (Figure 1). Moreover, it has deglycosylation activity similar to human NGLY1 [55]. The Suzuki group generated three microdeletion *Pngl* alleles (*Pngl*^*ex*14^, *Pngl*^*ex*18^, and *Pngl*^*ex*20^) through imprecise excision of a *P*-element insertion in the *Pngl* locus [54]. These *Pngl* mutant alleles behave as genetic null alleles and show developmental delay and semi-lethality, as less than 1% of homozygous and compound heterozygous animals were able to finish the pupal stage and emerge from the pupal cases as adult flies. The surviving adult flies are short-lived and sterile. These observations suggest a critical role for *Drosophila* NGLY1 in development and survival [54]. Rescue of the lethality phenotype of *Pngl* mutants by ubiquitous expression of wild-type mouse and human NGLY1 suggested functional conservation between *Drosophila* and mammalian NGLY1 [54,55].

Using the CRISPR/Cas9 gene-editing technique [56], the Perlstein group generated the *Pngl^PL^* allele to model NGLY1 deficiency [57]. This allele harbors a nonsense mutation at codon 420 and is predicted to disrupt the C-terminal carbohydrate binding domain. *Pngl^PL/PL^* animals showed developmental delay and reduced survival (only 32% of the pupae reached adulthood when reared at 25°C). *Pngl^PL/PL^* animals also displayed a small body size phenotype. Ubiquitous expression of human NGLY1 rescued the developmental delay and small body size phenotypes of these animals [57], indicating that the observed phenotypes are due to loss of *Pngl*, and further highlighting the functional similarity between Pngl and its mammalian homolog, NGLY1. In a study from the Chow group, ubiquitous knockdown (~95% reduction in gene expression) of *Pngl* also showed reduced survival (only ~30% of adult flies survived) and developmental delay [58]. In addition to the functional and structural similarity between *Drosophila* Pngl and mammalian NGLY1, consistent phenotypes exhibited by the independently generated *Drosophila* models for *Pngl* loss-of-function account for their utility to gain insight into the biological roles of NGLY1, as well as the pathophysiology of NGLY1 deficiency.

The *Caenorhabditis elegans* (*C. elegans*) homolog of NGLY1 is called PNGase 1 (PNG-1). It is encoded by *png-1*, which was identified by Kato and colleagues [59]. Instead of the PUB domain present at the N-terminus of fly Pngl and mammalian NGLY1, the worm PNG-1 contains a thioredoxin domain, which is capable of catalyzing protein disulfide reductase activity in vitro [5,59]. The first in vivo study on the function of the worm PNG-1 was performed by the Colavita group, who identified *png-1* loss-of-function alleles in a genetic screen for mutants that affect axon branching in the worm’s egg-laying organ [38]. The N-terminal thioredoxin domain and the PNGase catalytic domain were both required to rescue the axon-branching phenotype, but the C-terminal mannose-binding domain was not. The mutants showed abnormal egg-laying behavior as well, highlighting the functional consequence of the loss of *png-1*. A mutation in *png-1* was later shown to significantly reduce the worms’ lifespan [23]. As discussed in the following sections, studies in this model organism have provided the first in vivo evidence for the involvement of an NGYL1 homolog in the regulation of proteasome gene expression and mitochondrial function [23,60]. Moreover, the sensitivity of *png-1* mutant worms (and their fly counterparts) to proteasome inhibitors has been used in drug repurposing screens aimed to identify molecules with potential therapeutic benefits for NGLY1 deficiency [61]. We anticipate that studies in *C. elegans* will continue to provide important insight into the function of NGLY1 and will help identify additional proteins and pathways that are affected by the loss of *png-1* and/or can be manipulated to suppress the *png-1* loss-of-function phenotypes.

## 4. NGLY1 and the ERAD Pathway

### 4.1. NGLY1 Is a Component of the ERAD Pathway

As a part of the protein quality control mechanism, the ERAD pathway targets misfolded proteins from ER to cytosol for their degradation [62,63]. A number of human diseases, including cystic fibrosis and neurodegenerative and metabolic diseases, have been linked to ERAD dysfunction [64]. *N*-linked glycosylation of proteins in the secretory pathway facilitates their proper folding and trafficking, and processing of the *N*-glycan structures by specific glycosyl hydrolases is a critical step in determining which *N*-glycoproteins will be ERAD substrates and which can proceed through the secretory pathway [65]. A number of studies have used different model protein substrates to show a role for the deglycosylating activity of NGLY1 in ERAD [66,67,68,69,70]. In fact, the Cresswell group established a highly sensitive assay for ERAD by generating reporter proteins that only become fluorescent upon retrotranslocation from the ER and deglycosylation by NGLY1 [71]. Despite all these pieces of evidence, whether the deglycosylation activity of NGLY1 plays an essential role in ERAD remained to be seen. Ploegh and colleagues showed that inhibition of NGLY1 activity does not affect the proteasomal degradation of ERAD substrate glycoproteins like major histocompatibility complex (MHC) class I heavy chains [72], although it affected the presentation of glycoprotein epitopes by MHC class I [73]. Similarly, studies in a *Pngl* knockdown *Drosophila* model did not show a strong signature of ERAD dysfunction and ER stress [58]. Importantly, the Suzuki group used an isotope-coded glycosylation site-specific tagging (IGOT) system to identify several yeast glycoproteins that were deglycosylated by PNG1 in vivo and/or showed delayed degradation in a *png1* mutant strain [74]. This report demonstrated the yeast PNG1 as an ERAD component and established the IGOT method as a tool for the identification of NGLY1 substrates.

Histological analysis of *Ngly1^−/−^* rats revealed accumulation of cytoplasmic ubiquitinated proteins in neurons in the thalamus and spinal cord [46], suggesting ERAD dysfunction. This protein accumulation was found to be stronger in 5-week-old *Ngly1^−/−^* rats as compared to 29-week-old rats [46], suggesting that ERAD dysfunction might start at a young age in *Ngly1^−/−^* neurons. Impaired ERAD is often associated with ER stress. However, *Ngly1^−/−^* rat brains did not exhibit any difference in the level of ER stress markers eIF2, p-eIF2, GRP78, and XBP1, suggestive of no ER stress in this tissue [46]. Similarly, Asahina and colleagues demonstrated an accumulation of ubiquitinated protein in the thalamus, cerebral cortex, hippocampus, and spinal cord of the JF1/B6-*Ngly1^−/−^* mouse model [42], which is suggestive of an ERAD pathway defect. The authors found that ubiquitinated protein accumulation was prominent only in 5-week-old mice but not in 42-week-old mice. The reduction or lack of accumulation of ubiquitinated proteins at later ages as observed in *Ngly1^−/−^* rats and mice [42,46] suggests that a compensatory mechanism, presumably autophagy, might clear out the accumulated proteins in the CNS of aged rodents and subsequently reduce neurological defects. Of note, the adaptor protein p62 (official name: SQSTM1), which binds to ubiquitinated protein aggregates and directs them to autophagosome, was also found to be colocalized with ubiquitinated proteins in the brains of JF1/B6-*Ngly1^−/−^* mice [42]. Furthermore, a recent study reported that inhibition of NGLY1 by Z-VAD-fmk and siRNA-mediated *NGLY1* knockdown induce autophagosome formation in HEK293 cells [75]. The authors identified p62/SQSTM1 as one of the proteins enriched in autophagosomes upon NGLY1 inhibition or knockdown. In contrast, it has previously been suggested that at least for one substrate—ER degradation-enhancing α-mannosidase-like 1 protein (EDEM1)—deglycosylation by NGLY1 is required for EDEM1′s interaction with p62 and its targeting for autophagy [76], suggesting that NGLY1 is required for autophagy. Therefore, the precise role of NGLY1 in autophagy and its potential cell type-dependent differences remain to be determined. 

### 4.2. Engase Is A Modifier of NGLY1-Associated ERAD Dysfunction

Previous work by the Suzuki group identified ERAD defects in *Ngly1*-mutant MEFs and showed that these defects were improved upon simultaneous loss of the *Engase* gene [43]. In agreement with these observations, some *Ngly1^−/−^*; *Engase^−/−^* mice survived to adulthood with no noticeable phenotype at weaning age [39]. The double-mutant embryos did not show VSDs, anemia, or edema, suggesting that cardiovascular abnormalities are a major cause of embryonic lethality in *Ngly1^−/−^* mice and are rescued by loss of *Engase*. *Engase* encodes a cytosolic enzyme thought to be involved in the catabolism of free oligosaccharides, such as those released from *N*-glycoproteins by NGLY1 [77]. However, in the absence of NGLY1, ENGASE was able to act on cytosolic *N*-glycoproteins to release most of the *N*-glycan structure, leaving an unusual *N*-linked *N*-acetylglucosamine (*N*-GlcNAc) on the corresponding proteins. Importantly, presence of *N*-GlcNAc appeared to promote the formation of protein aggregates. Simultaneous removal of NGLY1 and ENGASE prevented the formation of such aggregates [43]. These data provided a likely mechanism for the suppression of *Ngly1^−/−^* phenotypes in double-mutant mice. It is worth mentioning that the surviving *Ngly1*^−/−^; *Engase*^−/−^ animals showed growth retardation and developed progressive neurological phenotypes over 6–7 months, with features including hindlimb clasping, front limb shaking, and impaired grooming suggestive of motor and coordination defects [39]. These observations indicate that some of the NGLY1 deficiency phenotypes arise independently from the function of ENGASE and the resulting *N*-GlcNAcylated proteins. Nevertheless, the available genetic and molecular data strongly suggest that inhibition of ENGASE might have potential therapeutic benefits for some NGLY1 deficiency phenotypes and have prompted efforts to identify ENGASE inhibitors [78].

### 4.3. The Ubiquitin Ligase Complex SCF^FBS2^ Mediates Proteasomal Dysfunction upon Loss of NGLY1 

A recent report by Yoshida and colleagues showed that simultaneous knockout of a sugar-recognizing F-box protein (FBS2; official name: FBXO6) rescued the lethality and motor functions in *Ngly1* knockout mice [79], suggesting that FBS2 makes a major contribution to *Ngly1* loss-of-function phenotypes. FBS2 and its homolog, FBS1, are cytosolic components of ERAD that recognize high-mannose glycans—usually found on misfolded *N*-glycoproteins—and promote the ubiquitination of such proteins, as well as their proteasomal degradation [80,81]. Notably, using unbiased proteomic analyses, the Steinmetz group recently reported FBXO6 downregulation in cell lines isolated from NGLY1-deficient patients compared to control cell lines, potentially as a protective mechanism against FBXO6-mediated toxicity in NGLY1-deficient cells [49]. The group has generated an excellent web-based application that can be used to examine whether a gene or protein of interest is differentially expressed in patient-derived cell lines compared to controls (https://apps.embl.de/ngly1browser accessed on 21 March 2022).

Because the glycan structure recognized by FBS2 is removed from the *N*-glycoproteins by NGLY1, loss of NGLY1 is predicted to generate new targets for FBS2 binding and their subsequent ubiquitination. Accordingly, Yoshida et al. used “nuclear factor erythroid 2-like 1” (NFE2L1; also called NRF1), as a well-known ERAD substrate and one of the direct targets of NGLY1 (see below), to examine this notion [79]. They showed that in the absence of NGLY1, the SFC^FBS2^ ubiquitin ligase ubiquitinates NFE2L1 and inhibits its nuclear transport. The ubiquitinated version of NFE2L1 in turn impairs proteasome function and leads to cellular toxicity. In *NGLY1^−/−^* cells, FBS2 overexpression exerts cytotoxicity and proteasomal inhibition. Importantly, this phenotype was rescued by a mutant version of NFE2L1 lacking *N*-glycans or by deletion of *NFE2L1*, indicating that the FBS2-mediated toxicity caused by ubiquitinated NFE2L1 is not due to loss of NFE2L1-mediated regulation of proteasomal gene expression. The precise mechanism for the toxic effect of ubiquitinated NFE2L1 on proteasome is not known. Nevertheless, because the *Fbs2*, *Ngly1* double knockout mice are healthy and viable and the *Fbs2* single knockout animals are viable without gross morphological defects, this study suggests a tantalizing therapeutic potential for FBS2 inhibitors for suppression of NGLY1-deficient patient symptoms.

## 5. Regulation of NFE2L1 Activation by NGLY1

### 5.1. NGLY1 Regulates NFE2L1-Mediated Proteasome Bounce-Back Response

**Figure 3:**
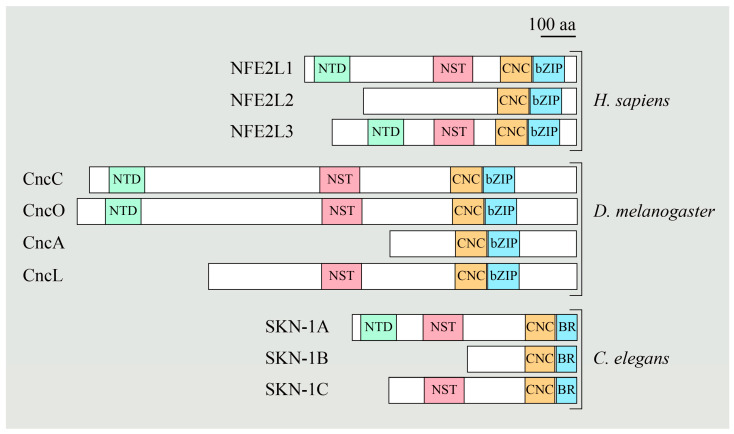
Schematic representation of the cap ’n’ collar proteins in the indicated species. Note that only representative isoforms of the fly cap ’n’ collar are shown. aa, amino acid; NTD, N-terminal domain; NST, Asn/Ser/Thr-rich domain; CNC, cap ’n’ collar; bZIP, basic leucine zipper; BR, basic region.

Impaired proteasome activity is implicated in a wide range of human diseases, including cancer, immune-related disorders, neurodegenerative diseases, and cardiac dysfunction [82,83]. Moreover, as the inhibition of proteasome activity has been shown to induce cell death [84,85], proteasome has received therapeutic attention as a drug target in cancer therapy [86]. Although several proteasome inhibitors, such as bortezomib (BTZ) and carfilzomib, have been approved for cancer therapy, development of resistance to proteasome inhibition limits the efficacy of these drugs. Proteasome inhibition is followed by a coordinated response aimed to restore the proteasome activity, which is called the ‘bounce-back response’ [87]. A transcription factor called NFE2L1, which is a member of the “cap ‘n’ collar” (Cnc) family, is essential for the upregulated expression of proteasome subunit (Psm) genes in mammalian cells [87,88]. A similar response of upregulation of Psm gene expression upon proteasome inhibition was observed in worms (*C. elegans*) and flies (*Drosophila melanogaster*) through the cap ‘n’ collar transcription factors SKN-1A and CncC, respectively [89,90]. Figure 3 shows a schematic representation of different cap ‘n’ collar family members in humans, *Drosophila*, and *C. elegans*. The NST (Asn/Ser/Thr-rich) domain of NFE2L1 contains seven putative *N*-glycosylation sites. Replacing asparagine (N) residues with glutamines (Q) decreased NFE2L1′s transcription activity, whereas replacing the asparagine (N) residues with aspartic acid (D) enhanced NFE2L1′s transcription activity [91]. It has previously been shown that de-*N*-glycosylation results in the conversion of the glycosylated N to D [92]. Therefore, these observations suggested critical roles for *N*-linked glycosylation and/or deglycosylation in NFE2L1 function. However, whether NGLY1 plays a role in NFE2L1 activation and proteasomal gene regulation remained unknown.

A key study in *C. elegans* by Lehrbach and Ruvkun [60] showed that the ER-association of SKN-1A (the worm homolog of NFE2L1) not only targets it for proteasomal degradation through ERAD but is also important for SKN-1A’s proper post-translational processing and activation upon proteasome inhibition. Using a forward genetic screen for genes required for SKN-1-mediated proteasome response, the authors identified mutations in ERAD components, an aspartic protease called *ddi-1*, and the worm homolog of *NGLY1*, which is called *png-1* [60]. This study proposed that glycosylated SKN-1A undergoes deglycosylation by PNG-1 after being released from ER and is then cleaved by the DDI-1 aspartic protease, a step required for the bounce-back response to proteasome inhibition. Koizumi and colleagues showed that in mammalian cell lines, de-*N*-glycosylation can occur even in the absence of DDI2 [93]. Regardless, this was the first study to report that an NGLY1 homolog is required for NFE2L1-mediated proteasome bounce-back response in vivo [60].

**Figure 4:**
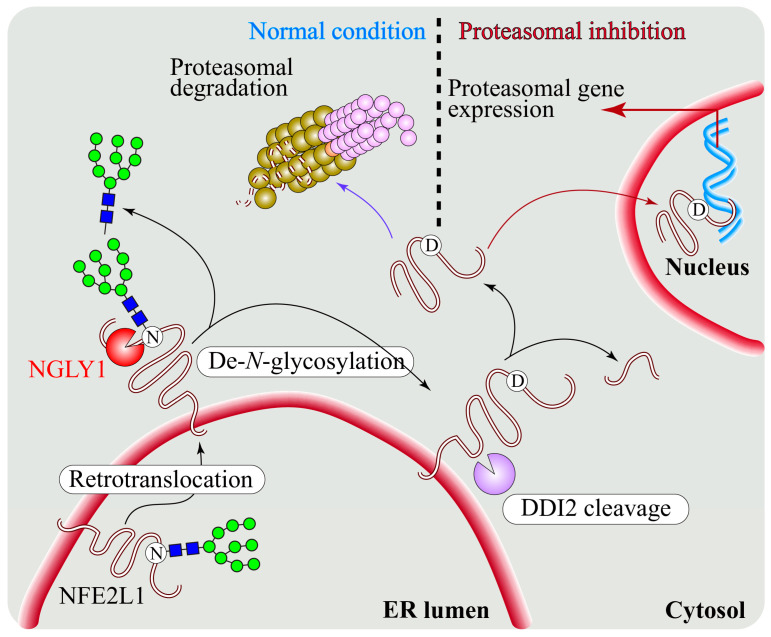
Schematic representation showing that under normal conditions, retrotranslocation from ER to cytosol directs NFE2L1 to its proteasomal degradation. Upon proteasomal inhibition, NFE2L1 undergoes de-*N*-glycosylation by NGLY1 and is cleaved by an aspartic protease called DNA damage-inducible 1 homolog 2 (DDI2), which leads to its activation. Activated NFE2L1 enters the nucleus and induces the transcription of proteasomal subunit genes, as well as its other target genes. N, asparagine; D, aspartic acid.

The importance of deglycosylation of mammalian NFE2L1 by NGLY1 was reported by the Bertozzi group [94]. They found that *Ngly1^−/−^* MEFs show accumulation of NFE2L1 at the ER, suggesting that DDI2 is unable to cleave the non-de-*N*-glycosylated NFE2L1 from the ER membrane. This suggested that NFE2L1′s de-*N*-glycosylation by NGLY1 is followed by its proteolytic cleavage and subsequent release from the ER (Figure 4), although the order in which NGYL1 and DDI2 operate on NFE2L1 is still not fully clear [94]. Importantly, genetic and pharmacological inhibition of NGLY1-mediated de-*N*-glycosylation led to NFE2L1 inactivation and impairment of the proteasome bounce-back response in mammalian cells. Moreover, a glycosylated NST domain might dampen the proper folding of NFE2L1 and subsequently incapacitate it to interact with Maf cofactors and DNA. Sha and Goldberg [95] reported that complete inhibition of 26S proteasome impairs the processing and transcriptional activity of NFE2L1, suggesting that the proteasome itself is involved in NFE2L1 cleavage. Later, Lehrbach et al. [96] reported that the conversion of *N*-glycosylated asparagine (N) residues to aspartic acid (D) residues in SKN-1A upon its de-*N*-glycosylation by PNG-1 is essential for the activation of SKN-1A in worms, showing for the first time that the N-to-D sequence editing of a target protein by NGLY1 plays a critical biological role.

The signature of NGLY1-NFE2L1-associated proteasome gene expression was also reported in *Drosophila* and in human leukemia cell line K562. In a study by the Chow lab, RNA sequencing analysis upon *Pngl* knockdown in adult flies revealed an enrichment of genes encoding for oxidoreductase and the genes encoding for the proteasome subunits in the downregulated pathway category [58]. Earlier, both of these gene categories were found to be downregulated upon CncC dysregulation in *Drosophila* [89,97]. Comparison with the gene expression data from a previous study showed that ~40 genes downregulated upon *Pngl* knockdown are targets of CncC [98]. Furthermore, a recent study from our group showed decreased expression of Psm genes in *Pngl* mutant larvae [99]. In fact, the fly Pngl appears to play such a critical role in proteasomal gene expression that even *Pngl* heterozygous larvae show enhanced sensitivity to BTZ [61]. Importantly, RNA sequencing and proteomic analyses on four different cell types from 14 patients with NGLY1 deficiency and 17 parent controls indicated the downregulation of multiple proteasome subunits in all examined patient cells [49]. Altogether, these studies provide compelling evidence that NGLY1 plays an evolutionarily conserved, essential role in the regulation of Psm gene expression by the NFE2L1 protein family. It is important to note that in addition to proteasomal gene expression, NFE2L1 serves several other key cellular functions, including promotion of mitophagy (see Section 6.2) [44] and providing resistance to a non-apoptotic form of cell death called ferroptosis [100], all of which seem to be impaired in NGLY1-deficient contexts. Therefore, whereas the focus of this section has been on impaired proteasome bounce-back response upon loss of NGLY1, disruption of other NFE2L1-mediated processes can also contribute to NGLY1 loss-of-function phenotypes in animal models and human patients. 

### 5.2. NFE2L1 Dysfunction and Pathogenesis of NGLY1 Deficiency

Some of the phenotypes associated with NGLY1 deficiency in human patients and mutant mice, including neurological and hepatic phenotypes and embryonic lethality, are similar to the phenotypes observed in mice with tissue-specific inactivation of the *Nfe2l1* gene [12,24,45,101,102,103,104]. These observations, combined with evolutionary conservation of the NGLY1-NFE2L1 axis, suggest that NFE2L1 dysfunction contributes to the pathogenesis of NGLY1 deficiency. However, the extent to which NFE2L1 dysregulation contributes to the NGLY1 deficiency phenotypes remains to be explored. In a recent report from our group, *Pngl* mutant *Drosophila* larvae showed decreased proteasomal gene expression, increased reactive oxygen species, and reduced ATP levels in their midguts [99]. However, restoring proteasomal gene expression in these larvae by feeding them with an NFE2L2 activator called sulforaphane did not rescue the oxidative stress and ATP levels in these animals [99]. Moreover, no abnormalities in the protein level of ER stress markers and NFE2L1-regulated proteasome subunits and deubiquitinating enzymes were observed by the Suzuki group in the brains of the *Ngly1^−/−^* rats discussed in Section 3.1, even though these animals showed developmental delay, movement disorder, and neurological symptoms similar to NGLY1-deficient patients [46]. In contrast, abnormal processing and cytoplasmic accumulation of NFE2L1 was observed in the livers of *Albumin-Cre; Ngly1^floxflox^* mice raised on a regular diet [45]. Moreover, as discussed in Section 4.3, accumulation of ubiquitinated NFE2L1 appears to play a major role in *Ngly1* loss-of-function phenotypes in mice [79]. Altogether, these observations indicate that some NGLY1 deficiency phenotypes are likely to arise from NFE2L1 misregulation. 

### 5.3. The NGLY1-NFE2L1 Axis as A Potential Therapeutic Target for Human Diseases 

NFE2L2 is a paralog of NFE2L1 that can recognize the *cis*-regulatory antioxidant response element (ARE) similar to NFE2L1. Unlike NFE2L1, NFE2L2 resides in the cytosol and is not *N*-glycosylated; therefore, it is independent of NGLY1 activity [105]. NFE2L2 regulates the cellular response to oxidative stress and also induces the expression of macroautophagy and proteasomal genes [106,107]. It also inhibits ferroptosis through a mechanism distinct from that of NFE2L1 [100]. Under normal conditions, Kelch-like ECH-associated protein 1 (KEAP1) retains NFE2L2 in the cytoplasm and directs it towards ubiquitination and proteasomal degradation [108]. Upon stress, NFE2L2 is dissociated from KEAP1 and dimerizes with the MAF protein on AREs to induce the expression of antioxidant enzymes [97]. Importantly, it has been reported that pharmacological activation of NFE2L2 by small molecules (including the KEAP1 inhibitor sulforaphane) can alleviate the mitophagic defects observed in *Ngly1^−/−^* MEFs [44]. To identify FDA-approved drugs that can suppress the BTZ sensitivity and larval growth defect caused by reduced NGLY1 activity, the Perlstein group used *Pngl^+/PL^* fly larvae and *Png-1^−/−^* worms treated with BTZ in parallel drug repurposing screens, followed by a more stringent assay in *Pngl^PL/PL^* larvae without BTZ treatment and by NFE2L2 activation assays in the human U2OS cell line [61]. These efforts led to the identification of three categories of compounds: NFE2L2 inducers, steroidal and non-steroidal anti-inflammatory drugs, and catecholamines/catecholamine receptor activators. It is worth mentioning that the atypical antipsychotic medication and dopamine receptor activator aripiprazole was found in both worm and fly screens and validated as an NFE2L2 activator in U2OS cells. Therefore, NFE2L2 inducers might provide therapeutic benefits for some NGLY1 deficiency phenotypes. Moreover, given the toxic effect of SCF^FBS2^-mediated NFE2L1 ubiquitination in *Ngly1^−/−^* mice, inhibition of SCF^FBS2^ and reduction of NFE2L1 levels might also offer attractive therapeutic avenues for NGLY1 deficiency. 

Inhibition of the ubiquitin-proteasome pathway is a well-established therapeutic strategy in some cancers (multiple myeloma and mantle cell lymphoma) and has the potential to serve as a therapeutic approach for other cancers [86,109]. However, proteasome inhibitors often cause significant side effects, some of which are due to off-target effects of these drugs [86]. Moreover, the NFE2L1-mediated proteasome bounce-back response is activated upon proteasomal inhibition, likely allowing the cells to develop some degree of resistance to these medications. As mentioned above, loss of NGLY1 blocks the activation of NFE2L1 and thereby renders animal models and mammalian cell lines highly sensitive to the toxic effects of proteasome inhibitors [60,61,94]. Accordingly, the Bertozzi lab hypothesized that NGLY1 inhibitors might further sensitize cancer cells to proteasomal inhibitors, thereby offering novel tools for combination therapy with proteasomal inhibitors [94]. To begin to test this hypothesis, they screened a library of cysteine protease inhibitor-like compounds for potential NGLY1 inhibitors and identified a candidate molecule, WRR139 [94]. They showed that WRR139 can inhibit NGLY1 function in vitro and enhance the effect of carfilzomib when used in combination. In a more recent study, Yu-Chieh Wang’s group reported that melanoma patient samples and cells lines express significantly higher levels of *NGLY1* compared to primary melanocytes [110]. Importantly, in vitro and in vivo experiments indicated that genetic and pharmacological inhibition of NGLY1 in melanoma cells exerts a strong anti-tumor effect, promotes apoptosis, and renders melanoma cells more sensitive to other chemotherapeutic agents, including bortezomib [110]. Together, these studies establish NGLY1 as a potential therapeutic target in several forms of cancer. The data further suggest that the combination of NGLY1 inhibitors and proteasome inhibitors may be used to broaden the scope of proteasomal inhibition as a strategy to treat cancer, as this would not only impair the proteasome bounce-back response in cancer cells but would also potentially allow oncologists to reduce the dosage of proteasomal inhibitors used in human patients and therefore help to reduce the off-target side effects of these drugs. 

## 6. Regulation of Energy Homeostasis and Mitochondrial Structure and Function by NGLY1

### 6.1. Mitochondrial Abnormalities in Patients with NGLY1 Deficiency 

A link between NGLY1 and mitochondrial abnormalities was first suggested by the Falk group based on a comprehensive meta-analysis of the transcriptome changes reported in multiple human mitochondrial respiratory chain diseases [111]. Around the same time, Enns and colleagues published the first detailed phenotypic characterization of eight patients with NGLY1 deficiency and noted that mitochondrial disorders are among the differential diagnoses commonly considered for these patients (along with Rett syndrome and neurotransmitter metabolism disorders) [11]. In fact, a number of NGLY1 deficiency phenotypes, such as developmental delay, seizures, elevated liver enzymes, muscle weakness, scoliosis, and chronic constipation, are frequently observed in mitochondrial disorders [11,12,24,25,28,112,113]. Liver and muscle biopsies from NGLY1-deficient patients showed structural and functional abnormalities in mitochondria [23,28]. Moreover, fibroblasts from NGLY1-deficient patients showed decreased basal and maximal oxygen consumption rates, which is indicative of abnormal mitochondrial function [28,99]. Together, these observations suggested a critical role for NGLY1 in the structural integrity and function of mitochondria. 

### 6.2. Evidence for Abnormal Mitochondrial Structure and Function in Animal Models of NGLY1 Deficiency

Mitochondrial phenotypes are observed in several NGLY1deficiency animal models, suggesting that the role of NGLY1 in mitochondria is evolutionally conserved. Studies in *Ngly1* null MEFs, *png-1* mutant worms, and *Pngl* mutant flies have shown structural and functional abnormalities in mitochondria [23,44,99]. The Nan group recently reported that *Ngly1^−/−^* MEFs exhibit impaired mitophagy, mitochondrial fragmentation, and inflammation [44]. They observed induction of interferon stimulating genes (ISGs) in *Ngly1^−/−^* MEFs. They discovered that the inflammation observed in *Ngly1^−/−^* MEFs is due to the activation of the DNA-sensing cGAS-STING pathway, which is stimulated by mitochondrial DNA released as a consequence of mitochondrial damage. Importantly, chronic activation of the cGAS-STING pathways was also found in *Ngly1^−/−^* mice, which were still-born lethal. Loss of STING in the germline did ameliorate the immune response activation in *Ngly1^−/−^* embryos but did not rescue their lethality [44]. This suggested that innate immune activation, although a component of NGLY1 deficiency, was not responsible for the observed embryonic lethality. It is worth noting that the above-mentioned study on the connection between NGLY1 and melanoma by Yu-Chieh Wang’s group indicated significant upregulation of type I and type III interferons, including *INFB1* and *IL29*, in human melanoma cells upon knockdown and pharmacological inhibition of NGLY1 [110]. The strong cytokine response induced by targeting NGLY1 in melanoma cells also contributed to the anti-melanoma effect triggered by NGLY1 inhibition. These observations further establish a critical role for NGLY1 in preventing aberrant immune activation in mammalian cells and indicate that immune hyperactivation contributes to the antitumor effects of NGLY1 inhibition in some contexts.

Impaired mitochondrial function and inflammation were previously reported in mice with adipose tissue-specific knockout of *Nfe2l1* [114]. These phenotypes overlap with the findings of increased immune response and mitochondrial damage in *Ngly1^−/−^* MEFs [44], suggesting that impaired NFE2L1 activation might contribute to mitochondrial abnormalities caused by the loss of NGLY1. Indeed, NFE2L1 was shown to regulate the expression of mitophagy genes downstream of NGLY1 [44]. The authors showed that overexpression of NFE2L1 or NFE2L1-ΔN (the transcriptionally active form of NFE2L1 that bypasses the requirement for NGLY1-mediated deglycosylation) or treating the cells with the NFE2L2 activator sulforaphane partially rescued mitochondrial fragmentation and elevation of ISG expression in *Ngly1^−/−^* cells. It is not clear whether these measures improved mitochondrial function as well. Nevertheless, impaired NFE2L1 activation is likely to contribute to mitochondrial abnormalities observed in NGLY1-deficient patients. In summary, although a specific phenotype in human NGLY1-deficient patients is yet to be directly linked to energy homeostasis defects, the presence of mitochondrial abnormalities in NGLY1-deficient model organisms strongly suggests that mitochondrial defects contribute to some aspects of the pathophysiology of NGLY1 deficiency.

### 6.3. A Conserved Role for NGLY1 in the Regulation of AMP-Activated Protein Kinase (AMPK) Signaling

AMPK is a highly conserved energy sensor that regulates cellular energy homeostasis [115]. It acts as a heterotrimer and comprises a catalytic subunit (AMPKα) and two regulatory subunits (AMPKβ and AMPKγ). When cellular ATP levels decrease and the ATP to AMP ratio is altered, AMPKα is phosphorylated by upstream kinases. The resulting phospho-AMPKα (pAMPKα) is itself a kinase that phosphorylates numerous downstream targets and thereby plays a profound role in multiple aspects of cellular energy homeostasis, including mitochondrial health and homeostasis [116]. AMPK promotes mitochondrial biogenesis in response to increased energy expenditure to produce more ATP [117]. Moreover, AMPK regulates mitochondrial homeostasis through mitophagy and mitochondrial fission [118].

We previously reported that *Pngl^−/−^* larvae display a severe failure to empty their gut, described as the food accumulation phenotype [55]. In a recent study, we sought to determine the mechanisms underlying this phenotype and found that it is associated with impaired gut peristalsis [99]. Moreover, the visceral muscle surrounding the intestine exhibited altered energy metabolism (decreased ATP and increased reactive oxygen species levels) and abnormal mitochondrial cristae in *Pngl* mutant larvae [99], consistent with mitochondrial abnormalities observed in other NGLY1-deficient models and muscles from NGLY1-deficient patients [23,28,44]. Interestingly, loss-of-function mutations in *Drosophila AMPKα* were previously reported to have impaired gut clearance due to reduced gut peristalsis [119], which is similar to the *Pngl^−/−^* phenotype. Genetic experiments showed that reduced mesodermal expression of *AMPKα* in *Pngl^−/−^* larval intestine is the cause of the food accumulation phenotype in these animals [99]. Remarkably, adding an extra genomic copy of *AMPKα* or overexpression of *AMPKα* in the visceral muscle not only rescued the mitochondrial abnormality and impaired energy homeostasis in the visceral mesoderm, but they also significantly improved the gut clearance in these animals and increased the survival from ~1% to 40–45%. These observations established that reduced *AMPKα* expression in visceral mesoderm leads to energy homeostasis defects in this tissue and significantly contributes to the lethality of *Pngl^−/−^ Drosophila* larvae. *Ngly1^−/−^* MEFs and NGLY1-deficient patient fibroblasts also exhibited reduced expression of *AMPKα1* and *AMPKα2* and impaired energy homeostasis, which were improved upon pharmacological enhancement of AMPK signaling [99]. These findings indicate an evolutionarily conserved role for NGLY1 in the regulation of *AMPKα* levels. It is worth mentioning that in *Pngl^−/−^* larval intestine, total AMPKα and pAMPKα protein levels are reduced at similar levels. However, the reduction in pAMPK levels in *Ngly1^−/−^* MEFs and NGLY1-deficient patient fibroblasts is more severe than the degree of reduction in AMPKα expression, suggesting that in addition to reduced *AMPKα* expression, loss of NGLY1 might also impair AMPKα phosphorylation in mammals.

### 6.4. Potential Mechanisms for the Regulation of AMPKα by NGLY1 

**Figure 5:**
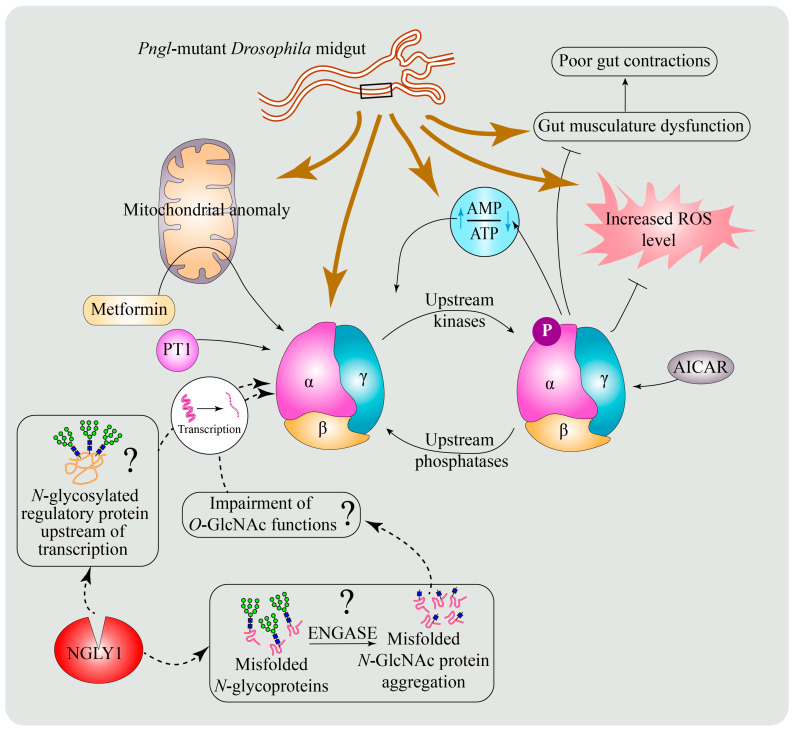
Schematic representation showing poor gut contraction in *Pngl*-mutant *Drosophila* midgut accompanied by reduced AMP-Activated Protein Kinase α (AMPKα) levels, increased AMP/ATP ratio, increased reactive oxygen species (ROS) levels, and mitochondrial anomaly. The cartoon also shows the AMPK activators metformin, PT1, and AICAR, as well as the possible mechanisms for regulation of *AMPKα* mRNA levels by NGLY1. NGLY1 might affect *AMPKα* transcription by altering specific upstream *N*-glycosylated proteins. In the absence of NGLY1, ENGASE may result in the accumulation and aggregation of proteins with *N*-GlcNAc residues, which might interfere with the function of proteins harboring *O*-GlcNAc. “?” marks potential mechanisms for the Regulation of *AMPKα* mRNA level by NGLY1.

The results from *Drosophila*, MEFs, and patient fibroblasts suggest NGLY1 acts upstream of AMPK signaling. However, it remains to be determined how NGLY1 regulates AMPKα levels. Our group examined the NGLY1-NFE2L1 axis as a potential mechanism. A reduction in proteasome gene expression and its rescue by sulforaphane treatment was observed in *Pngl* mutants [99], similar to what has been reported in *Ngly1^−/−^* MEFs [44]. However, sulforaphane treatment did not rescue *AMPKα* levels or the gut clearance phenotype in *Pngl* mutants. Similarly, despite rescuing the expression of proteasomal and mitophagy genes in *Ngly1^−/−^* MEFs, sulforaphane treatment did not result in any rescue of energy homeostasis defects in these cells. Lastly, loss of *Nfe2l1* and inhibition of proteasome function by MG132 did not lead to a reduction in AMPKα or pAMPKα levels in MEFs [99]. Together, these data demonstrated that the reduced AMPKα level upon loss of NGLY1 is independent of impaired proteasome function or NFE2L1 activity and suggested that loss of NFE2L1 activation is not sufficient to explain the energy homeostasis defects observed in *Ngly1^−/−^* MEFs. As discussed above, NGLY1 is a component of ERAD. Therefore, it is possible that loss of NGLY1 affects the quality control of any *N*-glycosylated cell surface receptor acting upstream of *AMPKα* transcription. Huang and colleagues demonstrated the cytosolic accumulation of an ERAD substrate with *N*-linked *N*-acetylglucosamine monosaccharides (*N*-GlcNAc) in *Ngly1^−/−^* MEFs [43]. Both *N*-GlcNAc and *O*-GlcNAc share similar epitopes. *O*-GlcNAc is the major glycan structure added to nucleocytoplasmic proteins and is involved in regulation of diverse processes, including transcription [120]. Therefore, given the similarity between *N*-GlcNAc and *O*-GlcNAc, it is possible that the appearance or aggregation of *N*-GlcNAc-linked proteins in the cytosol of NGLY1-deficient cells can impede the function of *O*-GlcNAcylated proteins involved in *AMPKα* transcription. Thereby, loss of NGLY1 might indirectly lead to reduced AMPKα levels through impaired *O*-GlcNAc function (Figure 5). Further work to explore these possibilities is likely to reveal a new regulatory mechanism linking deglycosylation to AMPK signaling.

### 6.5. Enhancing AMPK Activation as a Potential Therapeutic Strategy for NGLY1 Deficiency

AMPK is a druggable target. Medications such as metformin that indirectly activate AMPK have been used by humans for decades, and a number of AMPK activators and inhibitors are being developed by academic laboratories and the pharmaceutical industry for various diseases, including diabetes, metabolic syndrome, mitochondrial abnormalities, and neurodegeneration, as well as for research purposes [121,122,123,124,125,126,127]. The rescue of NGLY1 deficiency energy homeostasis phenotypes upon genetic and pharmacological increase in AMPK signaling in flies and mammalian cells, respectively, suggests that AMPK activators might offer a new strategy to treat some of the NGLY1 deficiency symptoms in patients. Considering that AMPK activators may have tissue preference [128], it would be important to determine the tissues and organ systems in mammals in which optimal AMPK signaling depends on the function of NGLY1.

## 7. Regulation of BMP Signaling by NGLY1

### 7.1. Role of De-N-Glycosylation in the Regulation of BMP Signaling

BMPs are members of the transforming growth factor beta (TGFβ) superfamily and are involved in animal development and multiple human diseases [129]. Newly synthesized BMP precursor proteins form inactive dimers in ER, which are cleaved to form active BMP dimers. Active dimers are secreted, bind to the receptors, and initiate signaling [130]. BMP ligands can signal both as homodimers and as heterodimers [130,131]. In vitro and in vivo studies have shown that BMP heterodimers have more signaling strength compared to homodimers [132,133,134,135,136,137]. BMP proteins and other members of the TGFβ family each harbor several predicted *N*-linked glycosylation sites and have been shown to be glycosylated in many cases [138,139,140]. *N*-glycans on these proteins play various roles, such as promoting ligand–receptor binding, maintaining the latent state of the ligand, and facilitating the balance between heterodimer and homodimer formation [139,141,142]. However, it was not known whether BMP proteins are ERAD substrates and whether de-*N*-glycosylation regulates BMP signaling. The first evidence for the role of de-*N*-glycosylation in BMP signaling was reported by our group in *Drosophila* [55]. Signaling mediated by Decapentaplegic (Dpp; the fly homolog of mammalian BMP4) regulates the development of two specific regions in the *Drosophila* larval intestine, called gastric caeca and acid zone, through phosphorylated Mothers against dpp (pMad) [143,144]. We found shortening of gastric caeca and loss of acid zone in the intestines of *Pngl*^*ex*14/*ex*14^ and *Pngl*^*ex*18/*ex*18^ third-instar larvae, phenotypes reminiscent of gut-specific loss of Dpp signaling [55,144]. This report demonstrated a requirement for the enzymatic activity of Pngl in the mesoderm to promote BMP/Dpp autoactivation in the visceral mesoderm and for proper intestinal development [55]. Of note, *Pngl* mutant *Drosophila* larvae showed a severe decrease in the level of Dpp homodimers, suggesting that de-*N*-glycosylation is essential for the formation and/or stability of Dpp homodimers [55].

**Figure 6:**
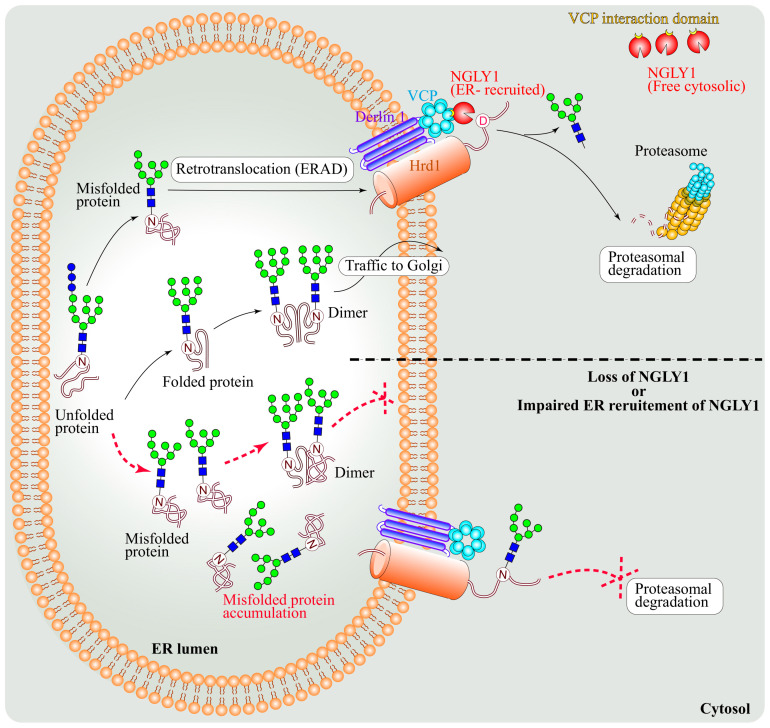
Schematic representation showing that de-*N*-glycosylation of misfolded bone morphogenetic protein 4 (BMP4) by endoplasmic reticulum (ER) membrane-recruited NGLY1 promotes its retrotranslocation from ER to cytosol, where it undergoes proteasomal degradation. In the absence of NGLY1 or its impaired ER recruitment, misfolded BMP4 molecules cannot be retrotranslocated to the cytosol and are accumulated in the ER lumen, where they can potentially dimerize with properly folded BMP4 molecules and prevent the trafficking of BMP4 dimers from ER to the Golgi. N, asparagine; D, aspartic acid; VCP, Valosin-Containing protein; ERAD, Endoplasmic reticulum associated degradation.

Further extending these findings, our group recently identified BMP4/Dpp as a direct target of NGLY1 in *Drosophila* and mammals and showed that NGLY1 functions in BMP4/Dpp retrotranslocation and signaling (Figure 6) [40]. In *Ngly1^−/−^* MEFs with BMP4^HA-Myc^ transfection, the level of pSMAD1/5 and the amount of secreted BMP4 homodimer were dramatically reduced compared to *Ngly1^+/+^* (control) MEFs. However, the mutant cells responded normally to exogenous BMP4, indicating that upon loss of NGLY1, BMP4 signaling is impaired in signal-sending cells but not in signal-receiving cells. Comparing the migration of full-length BMP^HA-Myc^ from cell lysate with or without digestion by PNGase or Endo H enzymes in SDS-PAGE gels revealed that BMP4 retains high-mannose (ER-type) *N*-glycans upon loss of NGLY1. A mutant version of BMP^HA-Myc^ with N-to-Q mutations in all of its four *N*-glycosylation sites was able to induce strong BMP4 signaling in MEFs. Remarkably, one copy of a knock-in allele of the *Drosophila dpp* with N-to-Q mutations in three of its four *N*-glycosylation sites fully rescued the BMP signaling defects in *Pngl^−/−^* intestines and partially rescued the lethality of the mutant larvae. Finally, staining of *Ngly1^−/−^* and control E15.5 mouse embryos with anti-pSMAD1/5 antibody showed a severe reduction in BMP signaling in several tissues, including the choroid plexus of the fourth brain ventricle and the heart [40]. Together, these observations strongly suggest that NGLY1/Pngl removes *N*-glycans from BMP4/Dpp to promote BMP signaling in both flies and mammals.

Staining with the ER marker KDEL showed that BMP4 is accumulated in the ER in *Ngly1^−/−^* MEFs [40]. Loss of NGLY1 led to upregulation of ER stress markers, such as GRP78/Bip (a major ER luminal chaperone) and pIRE1α (an indicator of unfolded protein response activation), in MEFs. Moreover, the level of an ER lectin called OS9, which binds to misfolded glycoproteins and is involved in their retrotranslocation, was increased in MEFs upon loss of NGLY1. In both control and *Ngly1^−/−^* MEFs, all of these phenotypes were further enhanced when BMP4 was overexpressed. Together, these data suggested that the ER stress observed in *Ngly1^−/−^* MEFs is due to the accumulation of misfolded BMP4 and potentially other misfolded proteins in the ER [40]. NGLY1 is a cytosolic protein thought to function after the retrotranslocation of misfolded proteins from the ER to the cytosol, and it has been assumed that loss of NGLY1 leads to the accumulation of ERAD substrates in the cytosol. Therefore, accumulation of BMP4 in the ER of *Ngly1^−/−^* MEFs and induction of ER stress by BMP4 in these cells suggested that this model might not be correct for all NGLY1 substrate proteins. Indeed, we found that upon accumulation of misfolded BMP4 in the ER, NGLY1 is recruited to the cytosolic side of the ER, likely via its protein–protein interaction with an ATPase called valosin-containing protein (VCP), which is recruited to the ER and functions in the retrotranslocation of misfolded proteins [8,145,146]. Importantly, upon introduction of point mutations in NGLY1 that block its interaction with VCP but do not impair its enzymatic activity, NGLY1 failed to be recruited to the ER and induce BMP4 signaling in *Ngly1^−/−^* MEFs. Finally, BTZ-induced proteasomal inhibition led to the accumulation of deglycosylated BMP4 in control MEFs, likely in the cytosol, but did not impair BMP4 signaling. Altogether, these data are compatible with the conclusion that NGLY1-mediated de-*N*-glycosylation of BMP4 is required for retrotranslocation of BMP4 and that although deglycosylated BMP4 ultimately undergoes proteasomal degradation, this step is not essential for BMP4 signaling [40].

### 7.2. Differential Regulatory Roles for NGLY1: BMP4 versus NFE2L1

Studying the regulation of BMP4 signaling by NGLY1 revealed several differences in the regulation of BMP4 versus NFE2L1 by NGLY1. First, only ER-recruited NGLY1 can deglycosylate BMP4, whereas ER recruitment of NGLY1 by VCP is not required for NFE2L1 deglycosylation, suggesting that NGLY1-dependent deglycosylation promotes the retrotranslocation of BMP4 but is not essential for the retrotranslocation of NFE2L1. Moreover, NGLY1 is required for the deglycosylation of misfolded BMP4 and directs it to proteasomal degradation, whereas NGLY1-dependent deglycosylation of NFE2L1 is required for its activation. The asparagine (N) harboring *N*-glycans in an *N*-glycoprotein is changed to aspartic acid (D) upon NGLY1-mediated deglycosylation [147]. The Ruvkun lab recently reported that NGLY1-dependent N-to-D sequence editing of *N*-glycosylation sites in SKN-1A (the *C. elegans* homolog of NFE2L1) is essential for its activation [96]. However, in the context of BMP4 signaling, N-to-D sequence editing is not required, as removal of *N*-glycans by NGLY1 is sufficient for the removal of misfolded BMP4/Dpp from the ER and promotion of BMP signaling by properly folded ligands.

### 7.3. Impaired BMP Signaling and Pathogenesis of NGLY1 Deficiency

NGLY1 deficiency affects multiple organ systems in human patients, including the CNS and the musculoskeletal system [11,12,14,37]. Moreover, ventricular septal defects were found to accompany the embryonic lethality in *Ngly1^−/−^* mice [39,40], and a VSD was recently reported in a patient with NGLY1 deficiency [35]. In addition, *Ngly1^−/−^* mouse embryos have small hearts with reduced myocardial trabeculae and also show a rudimentary, stump-like choroid plexus in the fourth ventricle [40]. Notably, we found impaired BMP signaling (based on reduced pSMAD1/5 levels) in certain tissues in *Ngly1^−/−^* mouse embryos, including the heart, the fourth ventricle choroid plexus, and the cerebellum [40]. Studies on mammalian models have shown that BMP signaling is required for the development of many organs, including the cardiovascular system, the nervous system, and the choroid plexus [129,148,149,150]. In the case of the choroid plexus, the Monuki lab showed that BMP4 is sufficient to generate choroid plexus epithelial cells from neuroepithelial progenitors [151]. It is worth mentioning that similar to *Pngl^−/−^ Drosophila* larvae, only some aspects of BMP signaling appear to be affected in *Ngly1^−/−^* mouse embryos. Nevertheless, the findings from fly and mouse models of NGLY1 deficiency, combined with cell culture studies, suggest a possible causative role for impaired BMP signaling in some of the NGLY1 loss-of-function phenotypes in human patients. The data also suggest that enhancement of BMP signaling might be beneficial to address some of the common issues seen in NGLY1-deficient patients, including osteopenia and poor bone health.

## 8. Other Biological Processes and Pathways Regulated by NGLY1

Despite their phenotypic variability, patients with NGLY1 deficiency do not appear to exhibit a clear genotype–phenotype correlation (Figure 2; Table 1; [11,12]). This suggests the involvement of genetic and/or environmental modifiers in determining the phenotypes displayed by each patient. Indeed, the extreme sensitivity of the *NGLY1* loss-of-function phenotypes to genetic background was shown in recent work from the Chow lab. By using a set of genetically heterogeneous *Drosophila* strains, they showed that depending on the genetic background, *Pngl*-knockdown flies show a wide range of survivability, from 100% lethal to fully viable [152]. Genome-wide association analysis on this panel identified a *Drosophila Pngl* modifier gene, *Ncc69*, which is the fly homolog of human *NKCC1* and *NKCC2* (official names: *SLC12A2* and *SLC12A1*, respectively) [152]. Ncc69/NKCC1 encodes for a member of the SLC12 family of Na^+^/K^+^/2Cl^−^ transporters. The authors showed that NKCC1 is *N*-glycosylated and that endogenous NKCC1 from *Ngly1* mutant MEFs migrates slowly in SDS-PAGE gel compared to that from wild-type MEFs. This suggested that in the absence of NGLY1, retention of *N*-glycans increased the molecular weight of NKCC1. Furthermore, the NKCC1 function was decreased in the absence of NGLY1 in MEFs. Notably, NKCC1 is widely expressed in secretory epithelia [153], potentially implicating impaired NKCC1 function in the lack of sweat and tears observed in NGLY1-deficient patients.

Another novel aspect of NGLY1 function was revealed in a study from the Freeze group, who showed transcriptional regulation of the water channel aquaporins (AQP) by NGLY1 [154]. The authors showed that NGLY1 regulates AQP expression levels at least in part through ATF1/CREB1 transcription factors. Interestingly, both *Ngly1^−/−^* and *Aqp1*-knockdown MEFs showed resistance to hypotonicity-induced lysis compared to wild-type MEFs. Impaired AQP expression might explain the lack of or reduction in tears commonly observed in NGLY1-deficient patients. It is worth noting that overexpression of an enzymatically inactive form of NGLY1 was able to revert the cell lysis resistance and increase *Aqp1* expression in *Ngly1^−/−^* MEFs as efficiently as overexpression of wild-type NGLY1 [154]. These observations strongly suggest that the enzymatic activity of NGLY1 is not required for its role in regulating AQP expression, unlike most other roles described for NGLY1 in cells and animal models. This is reminiscent of the role played by *Neurospora* PNGase—which lacks enzymatic activity—in the regulation of cell polarity in this organism [52] and suggests that some NGLY1 deficiency phenotypes might be independent of NGLY1′s deglycosylation activity. Table 2 summarizes the pathways and target proteins affected by NGLY1 deficiency.

## 9. Conclusions

In the eight years that have passed since the publication of the first cohort of patients with NGLY1 deficiency [11], our knowledge about the function of this gene has dramatically increased. Moreover, a strong intellectual and technical framework has been established to help move the field towards testing potential therapeutic approaches for this disease. NGLY1 deficiency case reports, work in invertebrate and rodent animal models, and biochemical and ‘omic’ studies have provided important insights into the biological functions of NGLY1 and are likely to establish the pathophysiology of specific NGLY1 deficiency phenotypes in the coming years. We have learned that there is broad variability in disease presentation and progression among patients, suggesting that an NGLY1 deficiency diagnosis should not be considered just in patients presenting with all of the classic disease phenotypes. A number of critical biological pathways have been identified as being regulated by NGLY1, including ERAD, proteasomal bounce-back response, BMP signaling, and mitochondrial homeostasis. Furthermore, model organism studies have led to the identification of biologically relevant targets of NGLY1 and genetic modifiers of *NGLY1* loss-of-function phenotypes, including ENGASE, NFE2L1, AMPKα, BMP4, STING, and FBS2. Judging by the unpublished data and preprints from multiple groups working on NGLY1, these lists are likely to significantly grow in the next 1–2 years. Most if not all of these targets and pathways are druggable, providing opportunities to establish novel therapeutic approaches for NGLY1 deficiency. Moreover, drug repurposing screens in invertebrate models and mammalian cells, including specific cell types differentiated from patient-derived induced pluripotent stem cells, can provide valuable therapeutic leads. The establishment of GlcNAc-Asn as a reliable proximal biomarker for the loss of NGLY1 is a critical step towards monitoring the therapeutic benefit of strategies such as gene therapy that aim to restore de-*N*-glycosylation activity in patients. Equally important will be the establishment of distal biomarkers that allow the facile assessment of disease severity and progress, as most therapeutic modalities are likely to reverse the disease process downstream of NGLY1 and without restoring de-*N*-glycosylation. The establishment of rodent models that recapitulate many disease phenotypes (including neurological deficits) has been another key step in the quest to develop a therapy for this disease. The improvement observed in the motor function and neuroinflammation of the *Ngly1^−/−^* rats by AAV9-mediated gene therapy has shown that at least some of the phenotypes caused by loss of *NGLY1* can be improved by restoring de-*N*-glycosylation after birth, generating significant hope and excitement for a gene therapy approach.

The remarkable progress made in NGLY1 research over the last 7–8 years is a cause for celebration. In our opinion, the roots of this rapid progress can be traced to several factors, including inspiring and resourceful patient families and the Grace Science Foundation (established by a patient family); the open and frequent exchange of unpublished data by many investigators involved in NGLY1 research; and the decades-long dedication of one scientist, Tadashi Suzuki, to understanding the function of this gene and generating numerous reagents and assays essential for NGLY1 research, a journey that started with his discovery of *N*-glycanase activity in mammalian cells (reported in 1993 [92]) and has not stopped.

We would like to finish this article by remembering the loss of several patients with NGLY1 deficiency in the last 2–3 years—a sobering reminder that there is still no approved therapy for this disease. When it comes to NGLY1 deficiency, the time has not yet come to rest on our laurels. 

## Figures and Tables

**Table 1:** List of patients reported in the literature and their phenotypes.

Reference	Age/ Sex	*NGLY1* Genotype	Phenotypes
Need et al., 2012 [14] Enns et al., 2014 [11] Lam et al., 2017 [12] Levy et al., 2022 [13]	12 y/M *	Q631Sfs/R401X	Developmental delay, movement disorder, hypotonia, seizures, EEG abnormalities, epilepsy, corneal ulcerations, liver fibrosis, microcephaly, dysmorphic features, small hands and feet, peripheral neuropathy, alacrima, elevated liver transaminases, constipation
Enns et al., 2014 [11]	5 y/M *	R401X/R401X	Developmental delay, epilepsy, intrauterine growth retardation, movement disorder, hypotonia, dysmorphic features, EEG abnormalities, seizures, alacrima, scoliosis, small hands and feet, elevated liver transaminases, constipation
Enns et al., 2014 [11] Lam et al., 2017 [12] Levy et al., 2022 [13]	9 y/M	R401X/R401X	Developmental delay, epilepsy, intrauterine growth retardation, movement disorder, hypotonia, seizure, corneal ulcerations, EEG abnormalities, alacrima, strabismus, elevated liver transaminases, scoliosis, constipation, liver fibrosis, small hands and feet, neuropathy
Enns et al., 2014 [11] Lam et al., 2017 [12]	4 y/F	R401X/R401X	Developmental delay, hypotonia, microcephaly, movement disorder, elevated liver transaminases, alacrima, strabismus, constipation, dysmorphic features
Enns et al., 2014 [11] Lam et al., 2017 [12]	18 y/F	R401X/R401X	Developmental delay, movement disorder, microcephaly, intrauterine growth retardation, hypotonic, EEG abnormalities, seizure, corneal ulcerations, alacrima, strabismus, elevated lactate, elevated liver transaminases, scoliosis, constipation, dysmorphic features, small hands and feet
Enns et al., 2014 [11]	9 m/F *	R401X/R401X	Developmental delay, microcephaly, hypotonia, movement disorder, intrauterine growth retardation, microcephaly, EEG abnormalities, seizure, alacrima, dysmorphic features
Enns et al., 2014 [11] Lam et al., 2017 [12] Levy et al., 2022 [13]	27 y/F	R458Kfs/R458Kfs	Developmental delay, intrauterine growth retardation, microcephaly, movement disorder, EEG abnormalities, alacrima, corneal ulceration, hypotonia, elevated liver transaminases, elevated lactate, peripheral neuropathy, constipation, scoliosis
Enns et al., 2014 [11] Kong et al., 2018 [23] Levy et al., 2022 [13]	11 y/F	R402del/R524X	Developmental delay, movement disorder, microcephaly, hypotonia, EEG abnormalities, alacrima/hypolacrima, strabismus, elevated lactate, elevated liver transaminases, constipation, small hands and feet, neuropathy
Caglayan et al., 2015 [24]	16 y/M *	N511Kfs/N511Kfs	Developmental delay, hypotonia, feeding problems, peripheral neuropathy, speech impairment, corneal ulcerations, alacrima
Caglayan et al., 2015 [24]	9 y/F	N511Kfs/N511Kfs	Developmental delay, movement disorder, hypotonia, EEG abnormalities, feeding problems, epilepsy, peripheral neuropathy, seizure, strabismus, speech impairment, dysmorphic features, elevated liver transaminase, scoliosis
Heeley et al., 2015 [25] Lam et al., 2017 [12] Levy et al., 2022 [13]	21 y/M	S116X/c.881+5G>T	Developmental delay, movement disorder, hypotonia, seizure, epilepsy, dysmorphic features, alacrima/hypolacrima, strabismus, elevated liver transaminases, liver fibrosis, constipation, scoliosis, neuropathy
Bosch et al., 2016 [26]	3 y/M	R401X/R401X	Developmental delay, movement disorder, alacrima/hypolacrima, hypotonia, peripheral neuropathy, microcephaly, strabismus
Lam et al., 2017 [12]	3 y/M	L318P/R390P	Developmental delay, movement disorder, feeding problems, EEG abnormalities, epilepsy, dysmorphic features, alacrima/hypolacrima, microcephaly, elevated liver transaminases, elevated lactate
Lam et al., 2017 [12] Levy et al., 2022 [13]	10 y/F	E311K/W244R	Developmental delay, movement disorder, feeding problems, EEG abnormalities, dysmorphic features, alacrima/hypolacrima, microcephaly, elevated liver transaminases, elevated lactate, neuropathy
Lam et al., 2017 [12] Kong et al., 2018 [23] Levy et al., 2022 [13]	11 y/M	W535X/L637X	Developmental delay, movement disorder, feeding problems, hypotonia, epilepsy, EEG abnormalities, dysmorphic features, alacrima/hypolacrima, microcephaly, elevated liver transaminases, elevated lactate, neuropathy
Lam et al., 2017 [12] Levy et al., 2022 [13]	13 y/F #	Q208X/c.930C>T (G310G; splice site)	Developmental delay, movement disorder, feeding problems, EEG abnormalities, epilepsy, dysmorphic features, alacrima/hypolacrima, microcephaly, elevated liver transaminases, elevated lactate
Lam et al., 2017 [12] Levy et al., 2022 [13]	15 y/M #	Q208X/c.930C>T (G310G; splice site)	Developmental delay, movement disorder, feeding problems, EEG abnormalities, epilepsy, dysmorphic features, alacrima/hypolacrima, microcephaly, elevated liver transaminases, elevated lactate
Lam et al., 2017 [12] Levy et al., 2022 [13]	22 y/F *	R401X/R401X	Developmental delay, movement disorder, feeding problems, EEG abnormalities, epilepsy, dysmorphic features, corneal ulcerations, alacrima/hypolacrima, microcephaly, elevated liver transaminases, elevated lactate, neuropathy
Van Keulen et al., 2019 [27] Panneman et al., 2020 [28]	9 y/F	Q613fs/Q613fs	Developmental delay, movement disorder, seizures, scoliosis, adrenal insufficiency
Chang et al., 2019 [29] Levy et al., 2022 [13]	7 y/F	R469X/R469X	Developmental delay, microcephaly, dysmorphic features, feeding problems, constipation, hypotonia, alacrima/hypolacrima, elevated liver transaminases, liver fibrosis, neuropathy, movement disorder
Haijes et al., 2019 [30]	18 y/M	c.247-2A>G/c.247-2A>G	Developmental delay, movement disorder
Haijes et al., 2019 [30]	26 y/F	c.247-2A>G/c.247-2A>G	Developmental delay, movement disorder, alacrima/hypolacrima, corneal ulcerations
Haijes et al., 2019 [30]	11 y/M	R586X/R586X	Developmental delay, intrauterine growth retardation, EEG abnormalities, alacrima, corneal ulceration, elevated liver transaminases, constipation, scoliosis
Haijes et al., 2019 [30]	6 y/F	R586X/R586X	Developmental delay, epilepsy, alacrima, EEG abnormalities, corneal ulceration, elevated liver transaminases, constipation
Haijes et al., 2019 [30]	15 y/F	L618X/Y539Gfs	Developmental delay, epilepsy, EEG abnormalities, intrauterine growth retardation, microcephaly, constipation, elevated liver transaminases, strabismus
Panneman et al., 2020 [28]	8 yr */?	R401X/C283W	Developmental delay, movement disorder, hypotonia, seizures, EEG abnormalities, peripheral neuropathy, dysmorphic features, small hands and feet, corneal ulceration, liver fibrosis, elevated liver transaminases, elevated lactate, epilepsy
Panneman et al., 2020 [28]	?/?	R401X/R401X	Developmental delay, hypotonia, peripheral neuropathy, dysmorphic features, small hands and feet, alacrima/hypolacrima, strabismus, elevated liver transaminases
Panneman et al., 2020 [28]	?/F	R401X/E356G	Developmental delay, movement disorder, hypotonia, seizure, EEG abnormalities, peripheral neuropathy, dysmorphic features, small hands and feet, strabismus, elevated lactate
Abuduxikuer et al., 2020 [20]	17 m/M	Y342C/R411X	Developmental delay, movement disorder, hypotonia, alacrima/hypolacrima, microcephaly, epilepsy, feeding problems, elevated liver transaminases, seizures, speech impairment
Abuduxikuer et al., 2020 [20]	5 y/F	Y342C/R411X	Developmental delay, movement disorder, hypotonia, alacrima/hypolacrima, epilepsy, microcephaly, feeding problems, elevated liver transaminases, constipation, seizures, speech impairment
Abuduxikuer et al., 2020 [20]	19 m/F	Y342C/R411X	Developmental delay, movement disorder, hypotonia, intrauterine growth retardation, epilepsy, alacrima/hypolacrima, feeding problems, microcephaly, elevated liver transaminases, seizures, speech impairment, dysmorphic features, elevated lactate
Abuduxikuer et al., 2020 [20]	8 m/F	S546Ffs/c.1003+3A>G	Developmental delay, hypotonia, intrauterine growth retardation, alacrima/hypolacrima, strabismus, microcephaly, small hands and feet, feeding problems, elevated liver transaminases, speech impairment, dysmorphic features
Abuduxikuer et al., 2020 [20]	4 y/F	S546Ffs/c.1003+3A>G	Developmental delay, movement disorder, hypotonia, intrauterine growth retardation, alacrima/hypolacrima, microcephaly, scoliosis, small hands and feet, feeding problems, elevated liver transaminases, speech impairment, dysmorphic features
Abuduxikuer et al., 2020 [20]	10 m/M	R328G/R328G	Developmental delay, movement disorder, hypotonia, intrauterine growth retardation, alacrima/hypolacrima, microcephaly, small hands and feet, feeding problems, elevated liver transaminases, constipation, peripheral neuropathy, dysmorphic features
Lipiński et al., 2020 [31]	7 y/M	c.1789+1G>A/c.1063T>C	Elevated liver transaminases, liver steatosis, global developmental delay, movement disorder, hypolacrima
Lipiński et al., 2020 [19]	1.5 y/?	E84X/R401X	Developmental delay, movement disorder, alacrima, elevated liver transaminases, hypotonia, hypolipidemia
Ge et al., 2020 [32]	10 m/F	R390X/D386Y	Developmental delay, intrauterine growth retardation, alacrima/hypolacrima, elevated liver transaminases, elevated lactate, EEG abnormalities, seizures, constipation
Rios-Flores et al., 2020 [33] Levy et al., 2022 [13]	8 y/M	Q631Sfs/N178Qfs	Developmental delay, movement disorder, hypotonia, intrauterine growth retardation, alacrima/hypolacrima, constipation, dysmorphic features, elevated liver transaminases, liver fibrosis, feeding problems, epilepsy
Lipari-Pinto et al., 2020 [34]	8 y/M	Q631Sfs/Q631Sfs	Developmental delay, hypotonia, elevated liver transaminases, small hands and feet
Kariminejad et al., 2021 [21]	30 y/M	W236C/W236C	Developmental delay, hypotonia, scoliosis, EEG abnormalities
Kariminejad et al., 2021 [21]	34 y/M	W236C/W236C	Developmental delay, hypotonia, movement disorder, seizures, scoliosis, constipation
Kariminejad et al., 2021 [21]	35 y/F	W236C/W236C	Developmental delay, hypotonia, scoliosis, constipation
Kariminejad et al., 2021 [21]	14 y/F	R390Q/R390Q	Developmental delay, movement disorder, epilepsy, liver fibrosis, EEG abnormalities, seizures, elevated liver transaminases, scoliosis
Kariminejad et al., 2021 [21]	29 y/F	R390Q/R390Q	Developmental delay, movement disorder, liver fibrosis, elevated liver transaminases, scoliosis
Stuut et al., 2021 [22]	5 y/M *	R401X/R401X	Developmental delay, alacrima/hypolacrima, movement disorder, epilepsy, intrauterine growth retardation, feeding problems, elevated liver transaminases
Dabaj et al., 2021 [35]	6.5 y/F *	R328C/R328C	Developmental delay, alacrima/hypolacrima, feeding problems, hypotonia, dysmorphic features, seizures, microcephaly, intrauterine growth retardation, elevated liver transaminases
Kalfon et al., 2022 [18]	6 m/F *	E432X/E432X	Developmental delay, feeding problems, hypotonia, alacrima/hypolacrima
Kalfon et al., 2022 [18]	3 y/M *	E432X/E432X	Developmental delay, hypotonia, movement disorder, EEG abnormalities, elevated lactate, elevated liver transaminases, peripheral neuropathy, seizure, feeding problems
Kalfon et al., 2022 [18]	12 y/F	E432X/E432X	Developmental delay, alacrima, movement disorder, hypotonia, EEG abnormalities, elevated lactate, elevated liver transaminases, seizure, feeding problems, peripheral neuropathy, microcephaly, scoliosis
Levy et al., 2022 [13]	17 y/F	N415Mfs/c.658+1G>A	Developmental delay, elevated liver transaminases, alacrima/hypolacrima, movement disorder, neuropathy, EEG abnormalities
Levy et al., 2022 [13]	15 y/M	R401X/deletion of at least exon 1–3	Developmental delay, elevated liver transaminases, alacrima/hypolacrima, movement disorder, neuropathy, EEG abnormalities, epilepsy
Levy et al., 2022 [13]	17 y/M	S169X/R383X	Developmental delay, elevated liver transaminases, alacrima/hypolacrima, movement disorder, neuropathy
Levy et al., 2022 [13]	8 y/M	S169X/R383X	Developmental delay, elevated liver transaminases, alacrima/hypolacrima, movement disorder, neuropathy
Levy et al., 2022 [13]	16 y/M *	R321X/Q631Sfs	Developmental delay, elevated liver transaminases, alacrima/hypolacrima, movement disorder, neuropathy, EEG abnormalities, epilepsy
Levy et al., 2022 [13]	4 y/M	R308W/c.1789+1G>T	Developmental delay, elevated liver transaminases, alacrima/hypolacrima, movement disorder, neuropathy, EEG abnormalities, epilepsy
Levy et al., 2022 [13]	7 y/F	R401X/c.1150-1G>C	Developmental delay, elevated liver transaminases, alacrima/hypolacrima, movement disorder, neuropathy, EEG abnormalities, epilepsy
Levy et al., 2022 [13]	3 y/F	R401X/c.1150-1G>C	Developmental delay, elevated liver transaminases, alacrima/hypolacrima, movement disorder, neuropathy, EEG abnormalities, epilepsy
Levy et al., 2022 [13]	6 y/F	R401X/C283W	Developmental delay, elevated liver transaminases, alacrima/hypolacrima, movement disorder, neuropathy, EEG abnormalities, epilepsy
Levy et al., 2022 [13]	5 y/M	R401X/deletion of intron3 and exon 3 splice junction	Developmental delay, elevated liver transaminases, alacrima/hypolacrima, movement disorder, neuropathy, EEG abnormalities
Levy et al., 2022 [13]	4 y/F	R401X/S607Ffs	Developmental delay, elevated liver transaminases, alacrima/hypolacrima, movement disorder, neuropathy, no EEG abnormalities
Levy et al., 2022 [13]	17 y/F	W369X/R469X	Developmental delay, elevated liver transaminases, alacrima/hypolacrima, movement disorder, neuropathy, EEG abnormalities, epilepsy
Levy et al., 2022 [13]	9 y/M	C355Y/R469X	Developmental delay, elevated liver transaminases, alacrima/hypolacrima, movement disorder, neuropathy
Levy et al., 2022 [13]	11 y/M	Q191X/Q191X	Developmental delay, elevated liver transaminases, alacrima/hypolacrima, movement disorder, neuropathy, epilepsy
Levy et al., 2022 [13]	9 y/F	Q631Sfs/Q631Sfs	Developmental delay, elevated liver transaminases, alacrima/hypolacrima, movement disorder, neuropathy, epilepsy
Levy et al., 2022 [13]	3 y/M	R401X/N511Kfs	Developmental delay, elevated liver transaminases, alacrima/hypolacrima, movement disorder, neuropathy
Levy et al., 2022 [13]	13 y/F	R469X/D597Sfs	Developmental delay, elevated liver transaminases, alacrima/hypolacrima, movement disorder, neuropathy, EEG abnormalities

* Deceased patients; ?, not reported; y, years; m, months; M, male; F, female; EEG, electroencephalography. Note that not all patient cohorts were examined for all the symptoms/phenotypes. The table summarizes only those phenotypes that were examined in the published reports. The siblings marked with # (first reported by Lam and colleagues [12]) exhibit significantly milder intellectual disability compared to other patients examined in that study, likely because the c.930C>T (G310G) splice site variant does not fully impair the splicing of the *NGLY1* mRNA and can lead to the expression of low levels of functional NGLY1 protein.

**Table 2:** List of the proteins and signaling pathways affected by NGLY1 deficiency in various model systems.

Associated Proteins or Signaling Pathways	Role of NGLY1	References
ERAD pathway	Deglycosylation of misfolded proteins	Hirsch et al., 2003 [67] Grotzke et al., 2013 [71] Hosomi & Suzuki, 2015 [68] Hosomi et al., 2016 [74]
BMP signaling (BMP4/Dpp)	Deglycosylation and retrotranslocation of misfolded BMP4/Dpp	Galeone et al., 2017 [55] Galeone et al., 2020 [40]
AMP kinase signaling and mitochondrial structural integrity	Regulation of *AMPKα* mRNA level	Han et al., 2020 [99]
Proteasomal homeostasis	Regulation of proteasomal bounce-back response by deglycosylation and activation of NFE2L1	Lehrbach et. al., 2016 [60] Tomlin et al., 2018 [94]
Resistance to hypotonic cell lysis	Non-enzymatic transcriptional regulation of aquaporin expression, in part through ATF1/CREB1	Tambe et al., 2019 [154]
Na^+^, K^+^, 2Cl^–^ ion transport	Regulation of NKCC1 function, potentially through NKCC1 deglycosylation	Talsness et al., 2020 [152]
Mitophagy and mitochondrial homeostasis	Regulation of mitochondrial biogenesis and mitophagy-related gene expression by deglycosylation and activation of NFE2L1	Yang et al., 2018 [44]
Innate immune signaling	Suppression of interferon signaling, at least in part through the DNA-sensing cGAS-STING pathway	Yang et al., 2018 [44] Zolekar et al., 2018 [110]
Melanoma survival and growth	Suppression of stress-signaling-associated apoptosis and cytokine surge	Zolekar et al., 2018 [110]
Ferroptosis	Resistance to ferroptosis through NFE2L1 deglycosylation	Forcina et al., 2022 [100]
